# Understanding How Alcohol Induces Human Acute Alcoholic Pancreatitis

**DOI:** 10.1016/j.ajpath.2025.03.008

**Published:** 2025-04-18

**Authors:** Isto Nordback, Hannu Paajanen, Stephen Pandol

**Affiliations:** ∗ Department of Gastroenterology and Alimentary Tract Surgery, Tampere University Hospital, Tampere, Finland; † Department of Gastrointestinal Surgery, University of Eastern Finland, Kuopio, Finland; ‡ Basic and Translational Pancreas Research, Cedars-Sinai Medical Center, Los Angeles, California

## Abstract

Why only a minority of heavy alcohol drinkers develop acute alcoholic pancreatitis has been a puzzle. In this review, the sparse data available from published studies were collected and, based on them, a hypothesis was formed. Long-term high alcohol consumption results in lowered cholecystokinin and cholinergic stimulus of the pancreas, and causes concentration and acidification of pancreatic fluid, predisposing to protein secretion. Early during the withdrawal period when returning to a normal or high-fat nonalcoholic diet, there is a relative hyperstimulation of the pancreas, a well-established mechanism that results in experimental acute pancreatitis. Lower, physiological stimulation is enough to start acute pancreatitis, when the secretions cause temporary obstruction in the duct system. The stimulation against temporary obstruction results in experimental acute pancreatitis. Finally, the magnitude of alcohol-induced deficits in acinar cell defense mechanisms determines the onset of pancreatitis.

Numerous studies have been undertaken to find out how various etiologies initiate acute pancreatitis. Crucial injury mediators would theoretically be important targets of treatment. However, because intracellular events occur mostly early in the course of pancreatitis, such treatments have usually failed in respective clinical studies. Therefore, there is currently no specific treatment for acute pancreatitis. However, improved supportive measures, including intensive care, together with less invasive treatments of late, local complications, have improved patient outcomes. Still, there is considerable mortality in this disease. In addition, recurrences may worsen long-term outcomes.^1^

The prevention of recurrent acute pancreatitis after the first episode is another aim of treatments. The two major causes of acute pancreatitis are gallstones and alcohol consumption. The simple measures to prevent recurrence are gallstone treatment and abstinence.^2^^,^^3^ Even though total abstinence prevents recurrences after the first episode of acute alcoholic pancreatitis, not all of those who continue with some alcohol intake develop acute recurrent pancreatitis.

The mechanism of how alcohol induces acute pancreatitis is unclear. An excellent short review of former theories was published a few years ago.^4^ Only a minority of those with high alcohol consumption may ever develop acute alcoholic pancreatitis. Therefore, other co-inciting or predisposing factors are required.

Smoking is clearly one contributing factors considered at least as important as alcohol intake in the development of chronic pancreatitis, but its role in triggering acute pancreatitis has not been shown.^5^ Genetics may also have some role, but the gene studies thus far may not explain more than some of the alcoholic cases.^6^ Several other factors have been studied, including helicobacteria, enteroviruses, and fatty acid profile, but without a causal connection.^7^, ^8^, ^9^

In this review, select findings on the early pathophysiology of acute pancreatitis are presented, and based on these, the essential initial steps in the pathophysiology of alcohol-induced acute pancreatitis in humans are suggested.

## Cellular Events in Acute and Chronic Pancreatitis

**Figure 1:**
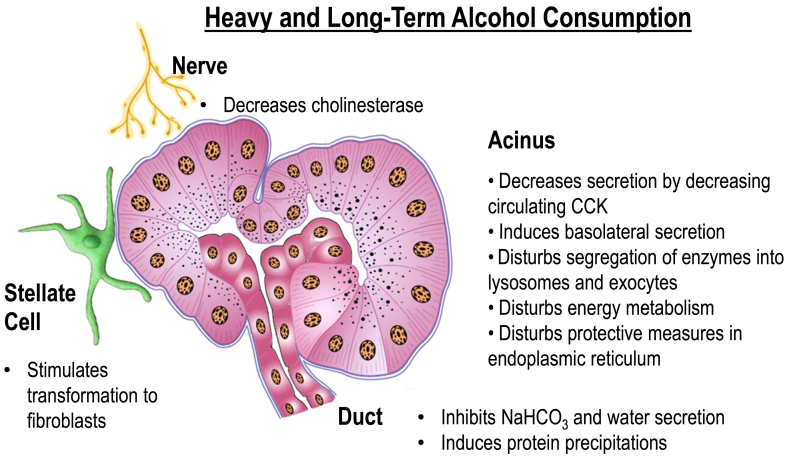
Heavy long-term drinking diminishes circulating cholecystokinin (CCK) levels, pancreatic cholinesterase activity, and possibly levels of muscarinic receptors. Alcohol use may also decrease the protective redirection of harmful proteins for lysosomal degradation in autophagosomes for later use, and may predispose to basolateral exocytosis after a physiological secretion stimulus. Alcohol stimulates stellate cells to transform fibroblasts. The effect of alcohol on the function of cystic fibrosis transmembrane regulator in ductal epithelium is to decrease the output of water and bicarbonate into the ductal fluid. This low-flow state and acidification may predispose to higher CCK release in the duodenum after stimulus. These changes are slightly deleterious, but do not induce acute pancreatitis during alcohol consumption. Reproduced with permission from Springer Nature.^11^

**Figure 2:**
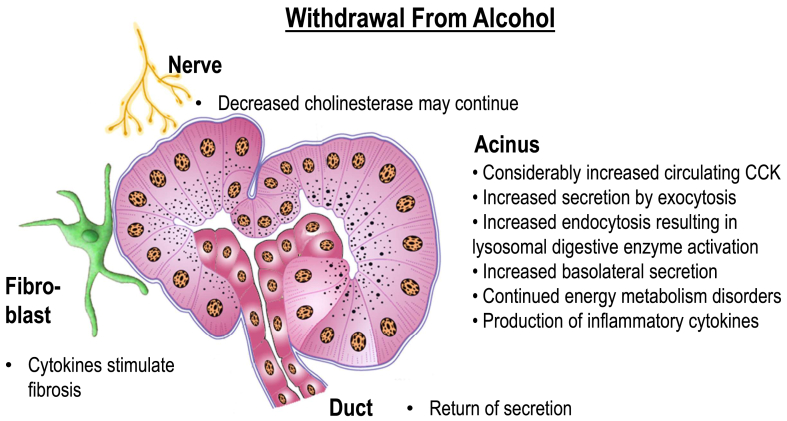
Cessation of alcohol intake and return to alcohol-free diet increases cholecystokinin (CCK), which together with decreased cholinesterase activity, induces high stimulus and secretion. The secretion may also be directed toward deleterious basolateral membrane. Increased secretion via exocytosis both basolaterally and apically may bring some secreted enzymes back into the cell via endocytosis, which is important in maintaining the normal size of the outer cell membrane. When this occurs, the capacity of autophagy is overwhelmed because of prevailing failures in the rough endoplasmic reticulum and in the Golgi apparatus, and some cellular structures are damaged. These, together with deleterious changes in mitochondrial metabolism, trigger inflammation with paracrine and endocrine spread. Even milder stimulus suffices to start the cascade if there is a blockade against which the cells try to secrete. Endocytosis may thus increase. In that case, such blockage may be caused by concentrated pancreatic fluid with precipitation or by concentrated bile with sludge. The latter is called “bile sludge–mediated alcoholic pancreatitis.” Reproduced with permission from Springer Nature.^11^

A recent review summarized well the intracellular events in the acinar cells, which have been considered the heart of acute pancreatitis, because of their high content of potentially injurious enzymes.^10^ Simplified schematics of these events are presented in Figures 1 and 2.^11^ There is strong evidence that the activation of proteases and lipases results in autodigestion of some important cell organelles, thereby injuring the acinar cells and initiating the proinflammatory cascade. Mitochondrial dysfunction participates in the injury, resulting in cell death.^12^^,^^13^

In healthy cases, zymogen granules containing the pro-forms of the proteases (preproteases) exocytose and merge with the cellular apical membrane, thereby releasing the contents into the lumen. The exocytosis occurs by the outer cellular membrane forming intracellular granules via endocytosis, which also prevents unreasonable enlargement of the cell membrane. These intracellular granules (endocytes) may contain some pre-protease molecules, especially in the case of hyperstimulation or physiological stimulation with duct obstruction, because they are abundant in the nearby apical lumen. These endocytes are then colocalized with lysosomes, forming large intracellular vacuoles. Lysosomal enzymes may degrade proteases into active forms, resulting in nonphysiological degradation of organelles.

Endocytosis and degradation of its content is also a physiological phenomenon for obtaining material to be reused in cellular structure and function. If this autophagy fails, the autophagosome-containing products do not degrade to smaller polypeptides or amino acids, and their enzymes remain in active form. Therefore, failure in the mechanisms of autophagy may be important in the initiation of pancreatitis.^10^

Mechanical injury of the acinar cells also results in pancreatitis. It is notable that this injury spreads from the initial site of injury.^14^ A direct tissue injury initiates the proinflammatory cascade, the intensity of which is dependent on the richness of acini.^15^

Other mechanisms of injury resulting in acute pancreatitis include the loss of cellular integrity and vitality when infusing substances into the pancreatic duct with high pressure.^16^ If the infused substances are toxic to the cell membrane, such as unconjugated bile acids or some contrast media during endoscopic retrograde cholangiopancreatography (ERCP), their infusion, even at low pressure, may result in cellular injury and pancreatitis.^16^^,^^17^ Furthermore, temporary ischemia appears to be a mechanism of initiating the acinar cell injury.^12^ Thus, any event that damages the acinar cells in sufficient amounts results in acute pancreatitis.

Injury also initiates recovery mechanisms, which is a phase of scar formation. Inflammatory cytokines induce, primarily via transforming growth factor β, pancreatic stellate cells and circulating stem cells attracted into the pancreas to transform fibroblasts. These cells synthesize collagen and other extracellular matrix components.^18^ Fibrosis that surrounds leaked fluid accumulations form pseudocysts. Overall, pancreatic fibrosis may induce narrowing of the pancreatic duct or ductules and replace destroyed acinar structures. This is how chronic pancreatitis develops after acute pancreatitis. Scar-obstructed duct may induce high pressure, which, together with scarring around the neurons, may result in chronic pain. The secretory capacity, either by diminution of functioning acini or by duct obstruction, may result in clinically significant exocrine insufficiency.

Conclusion: Although the cascade summarized above is quite well-known, the initial mechanism of alcohol-induced acute pancreatitis is not that well understood.

## Effects of Alcohol and Its Metabolites on the Pancreas

Alcohol or any of its metabolites alone have not been reported to cause acute pancreatitis in any experimental setting.^4^ Still, ethanol, as well as its oxidative and nonoxidative metabolites, are toxic to acinar cells in humans.^19^ Recently, attention has been focused on the mode of consumption and the amount of alcohol consumed in experimental models of pancreatitis. Binge drinking for a period of 10 days alone, or for 3-day periods together with background continuous low-dose alcohol use, may induce oxidative stress in endoplasmic reticulum, vacuolization, and some induction of cytokines.^20^^,^^21^ Smoking, another important factor in chronic pancreatitis, probably via the effects on stellate cells, is well-known for its capacity to induce oxidative stress. Furthermore, ethanol induces or sensitizes satellite cells leading to the production of collagen by fibroblasts.^22^ These mechanisms may explain how alcohol induces chronic pancreatitis without clinically detected acute pancreatitis episodes. Also, further understanding of such phenomena at the molecular level may offer possibilities for the development of therapies that discontinue the progression of the disease. However, this does not appear to be the mechanism that triggers acute alcoholic pancreatitis.

In addition to high consumption of alcohol, a combined factor is required to mimic initiation of clinical acute pancreatitis. In one study, short-term ischemia before acetaldehyde exposure induced edema, hyperamylasemia, and cellular injury in an isolated canine pancreas preparation, whereas short ischemia with ethanol or with acetate, the end-product of oxidative ethanol metabolism, had no effect.^23^ Polymorphism of alcohol dehydrogenase enzyme subtypes may increase the risk for acute pancreatitis, supporting the role of acetaldehyde.^24^ However, the existence of a variant of acetaldehyde that diminishes the oxidative metabolism of ethanol does not explain the development of acute alcoholic pancreatitis, except for in a minority of patients. There is no evidence that ischemia is the preceding or co-inciting factor in human acute alcoholic pancreatitis, prompting a search for other triggers.

Blocking of the oxidative metabolism of ethanol results in nonoxidative metabolism to fatty acid ethyl esters (FAEEs) in the pancreas with vacuolization and increased trypsinogen activity, as seen in acute pancreatitis.^25^ Induction of inflammation has been observed in human pancreatic tissue homogenates exposed to FAEEs.^26^ One must note, however, that this type of handling results in both tissue trauma and at least some ischemia, which may be important cofactors in inducing injury in such experiments. These findings do not explain why only a minority of alcoholic patients develop acute pancreatitis. Also, the formation of FAEEs requires significant failures in oxidative ethanol metabolism, and patients who fail abstinence during the disulfiram treatment (inhibitor of aldehyde dehydrogenase) do not usually experience acute pancreatitis. Thus, the role of FAEEs in the triggering of clinical acute alcoholic pancreatitis alone is unlikely.

Conclusion: Alcohol or any of its metabolites alone do not explain the onset of alcoholic pancreatitis.

## Alcohol- and Hyperstimulation-Induced Pancreatitis

Korsten et al^27^ reported that physiological stimulation of the pancreas by cholecystokinin, acetylcholine, or secretin was harmless. On the other hand, supramaximal stimulation by a cholecystokinin analogue, cerulein, induced acute pancreatitis, the severity of which was not increased by preceding alcohol ingestion. Cholecystokinin receptor 1 agonist induces pancreatitis in primates,^28^ and low-dose cholecystokinin-mediated exocytosis is redirected by ethanol from apical to basolateral membranes.^29^ This may be considered one marker of early injury but can be tolerated if low in quantity. So, although supramaximal stimulation can not be exaggerated by alcohol, physiological stimulation can.

Similar to that by cholecystokinin, hyperstimulation of pancreatic muscarinic receptors induces acute pancreatitis, which is also not exaggerated by preceding alcohol ingestion.^30^ In incubated tissue explants, supramaximal stimulation obtained with acetylcholine and cholinesterase inhibitors results in vacuolization and basolateral exocytosis as well.^31^ In an earlier study, long-term alcohol ingestion decreased the number of muscarinic receptors but did not affect the affinity of the ligand to the receptor.^32^ Thus, a theory of relative hyperstimulation by increased postalcoholic muscarinic tone was proposed.^33^ Also in humans, a scorpion sting induced acute pancreatitis probably via cholinergic hyperstimulation. Although this scorpion sting etiology is extremely rare, it is a good example of hyperstimulation initiating acute pancreatitis.^34^

The effects of cholecystokinin have been studied in human pancreatic slices ex vivo. Physiological dose induces apical exocytosis, while supramaximal doses induces redirection of exocytosis to the basolateral plasma membrane, autophagy, and increased trypsin activity. The effect is mediated via cholecystokinin receptor type A (or 1) and not via the cholecystokinin receptor type B (or 2). These are the receptors in the acinar cells and not in the cholinergic neurons because the effect could not be ameliorated with atropine.^35^

Conclusion: Hyperstimulation of the pancreas by cholecystokinin or by acetylcholine is injurious to acinar cells, initiating acute pancreatitis. Alcohol does not potentiate hyperstimulation-induced pancreatitis.

## Acute Pancreatitis Induced by Stimulation against Obstruction

Before delving into the initial steps after injury in acute alcoholic pancreatitis, one must understand the initial mechanisms of biliary and post-ERCP pancreatitis, because acute pancreatitis is similar to acute alcoholic pancreatitis, independent of the etiology.

When a person eats, duodenal cholecystokinin, together with vagal cholinergic stimulus, contracts the gallbladder and pushes possible gallstones or sludge that may obstruct the distal end of the bile and pancreatic ducts downstream. In suitable temporary pressure circumstances, bile may also reflux into the pancreatic duct, potentiating injury. The injection of various bile acids into the pancreatic duct induces acute pancreatitis.^36^ Also, the presence of gallbladder in patients has been associated with more severe pancreatitis compared to patients without a gallbladder, suggesting a role for bile in promoting pancreatitis severity.^37^ Bile reflux–initiated acute pancreatitis may be, at least in part, mediated by G protein coupled bile acid receptor 1 in pancreatic acinar cells, affecting calcium signaling, which in turn is important in acinar cell secretion.^38^ Recent studies have suggested other G protein–linked receptors as being the mediators.^39^ Regardless, it appears that bile acids are not only toxic to cell membranes but may also enhance acinar cell secretion when in contact with them.^40^

In the opossum, the reflux of bile aids into the pancreatic duct is not necessary for acute pancreatitis, and acute pancreatic duct obstruction alone may well suffice.^41^ However, in humans, where pancreatic duct obstruction may develop slowly by a tumor distal from papilla, acute pancreatitis is not a common result, although it is a known consequence. Also, bile duct obstruction above the papilla level alone does not induce acute pancreatitis. Furthermore, pancreatic stone blockage of the main pancreatic duct in chronic pancreatitis is often not accompanied by acute pancreatitis. This may be because, in both cases, the pancreas is already induced to produce protective scarring (fibrosis), in which case the proinflammatory reaction is diminished.^15^

In rats, bile duct obstruction alone rapidly increases circulating cholecystokinin levels. These levels are further enhanced when both the pancreatic and bile ducts are acutely obstructed.^42^ This is because bile and pancreatic fluids in the duodenum exert a negative-feedback response to cholecystokinin release.^43^ This is, at least in part, mediated by the neutralization of the acidic duodenal pH by alkaline bile and pancreatic fluid.^44^ Cholecystokinin levels can be lowered by somatostatin, which depresses the duodenal cholecystokinin–releasing peptide.^45^ Blocking of the acini by both somatostatins, which decrease cholecystokinin levels, and cholecystokinin receptor, ameliorates experimental obstruction-induced pancreatitis.^46^^,^^47^

Sphincter of Oddi spasm induced by cannulation is important in triggering post-ERCP pancreatitis. This may occur even when contrast medium has not been injected into the pancreatic duct. However, prorelaxing drugs such as nifedipine and glyceryl trinitrate have been ineffective in preventing this complication.^48^ Cannulation of the tight papilla induces temporary obstruction. In fact, the difficulty, and thus the duration, of cannulation is a known risk factor for post-ERCP pancreatitis. In a study in patients with post-ERCP pancreatitis, the cholecystokinin levels were increased substantially from pre-ERCP levels within hours, followed by a marked decrease during the first 24 hours after ERCP, possibly caused by duodenoscopy irritation and gas blowing.^49^ Similarly, in patients with gallstone and alcoholic pancreatitis, where pre-illness or very early samples cannot be obtained, decreased values were observed during hospitalization.^50^ Thus, an acute obstruction increases circulating cholecystokinin levels that may be important in the very early pathogenesis of acute pancreatitis in post-ERCP and in biliary etiology. This is supported by the finding that the inhibitors of secretion, cholecystokinin antagonist and somatostatin, ameliorate pancreatitis when introduced early in experimental settings.^51^ In humans, such blockers may be given early to prevent post-ERCP pancreatitis. In meta-analyses, somatostatin has been found to be effective.^48^ Understandably, it does not have an effect on patients with alcoholic and biliary pancreatitis, admitted to hospital, already with progressive disease.^52^

Conclusion: Physiologic stimulation may suffice to induce acute pancreatitis in case of simultaneous acute pancreatic duct obstruction. Such stimulus may be enhanced with simultaneous bile duct obstruction, and further by refluxed bile into the pancreatic duct.

## Other Mechanisms Induce Acute Pancreatitis

In addition to the mechanisms listed in the previous paragraphs, the secretion of fluids induced by secretin against partial obstruction is also deleterious to pancreas. Furthermore, ischemia and reperfusion, short-term ischemia plus acetaldehyde infusion, and free fatty acid infusion all result in changes consistent with pancreatitis. The morphologic changes in stimulation models (cholecystokinin analogue cerulein and secretin against partial obstruction) were predominantly in the acinar cells or ducts, whereas in the models of free fatty acid infusion, ischemia plus reperfusion, and short-term ischemia plus acetaldehyde models, the injury initiated from the intrapancreatic vessels. In the vascular- and duct-predominated models, the oxygen free radicals seemed to be important injury triggers, whereas free radical scavengers were ineffective in the other models.^53^ Except in the most severe model (free fatty acid infusion), anaerobic glucose metabolism maintained ATP levels.^54^ Of the five models with isolated canine pancreas preparation, a cholecystokinin receptor blocker ameliorated hyperstimulation with cholecystokinin analogue cerulein.^55^^,^^56^ Therefore, besides the effects on secretion, effects on energy metabolism may be important in mediating the injury, independent of the mechanism of initiation of acute pancreatitis.

Direct surgical trauma may induce injury that spreads throughout the pancreas.^14^^,^^57^ This injury then initiates a proinflammatory cascade. Such a proinflammatory response is diminished if acinar tissue is decreased and fibrosis is increased in the pancreas.^15^ Pretreatment with corticosteroids may ameliorate this injury response.^58^

Conclusion: In addition to hyperstimulation and stimulation against obstruction, pancreatitis may be initiated also with bile salt infusion, high intraductal pressure, ischemia, and direct trauma. Changes in energy metabolism followed by proinflammatory triggers are important early mechanisms.

## Is preceding Chronic Pancreatitis Necessary before Acute Alcoholic Pancreatitis?

Patients with alcohol-induced acute pancreatitis fall in either of the two categories: those with detected chronic pancreatitis with an acute phase and those with the first diagnosed acute alcoholic pancreatitis without any findings of previous chronic pancreatitis.

In chronic alcoholic pancreatitis, there is a decrease in acinar tissue and marked fibrosis, together with irregularities or even obstructions in ducts. Decades ago, this was thought to be due to the induction of pancreatic lithostatic protein by alcohol, secreted into the pancreatic fluid and increasing the viscosity, thereby forming protein plugs obstructing ductuli.^59^ Later, the effects of alcohol on ductal cell cystic fibrosis transmembrane conductance regulator (CFTR) were shown to promote acute pancreatitis; CFTR function was restored with a substance called orkambi to ameliorate the severity of pancreatitis.^60^ These findings may mean that acidification and concentration of pancreatic fluid that occurs when CFTR function deteriorates might induce secretion blockages in ductules.^61^

Several factors promote fibrosis by inducing stellate cells. These include alcohol and cigarette smoke, known etiologic factors of chronic pancreatitis. Mechanical stress and acinar cell injury, together with inflammatory cells, induce stellate cells.^62^ Thus, there is a possibility of chronic pancreatitis preceding the acute phase even before the diagnosis of chronic pancreatitis. However, in the long term, after the first episode of acute alcoholic pancreatitis, almost half of patients have recurrent pancreatitis episodes. Of those, almost 90% develop diabetes, and 25% develop exocrinic insufficiency.^63^ At 7 years after the initial episode, 90% of patients have morphologic findings of chronic pancreatitis.^64^ These rates were considerably lower among patients without acute recurrences during follow-up.^65^ Thus, many patients develop clinical manifestations of chronic pancreatitis after, not before, the first episode of acute alcoholic pancreatitis.

Although the effects of alcohol on pancreatic fluid contents and on stellate cells may provide insight into how chronic pancreatitis initiates, these findings do not explain the trigger mechanism of acute alcoholic pancreatitis. The slow-developing increased viscosity of pancreatic fluid, together with protein plugs, would need a coinciding stimulus, as explained in the previous sections. Scarring due to previous chronic pancreatitis may limit the spread of inflammation, as is the case in pancreatitis after pancreas surgery.^15^^,^^66^ In fact, mild alcoholic pancreatitis, possibly also due to protection of preceding undiagnosed chronic pancreatitis, recurs more often and consequently more often results in clinical chronic pancreatitis.

Conclusion: Preceding chronic alcoholic pancreatitis may predispose to fluid protein plugs, which, in the case of physiological stimulus, may result in acute pancreatitis. The preceding chronic pancreatitis also may have a protective role in the limitation of any acute attack.

## Clinical Observations in Patients with Acute Alcoholic Pancreatitis

To understand which of the mechanisms described in the preceding sections most likely play a role in the initiation of human acute alcoholic pancreatitis, one needs to refer first to the clinical findings of human acute alcoholic pancreatitis.

In a study from Germany, 1-week heavy alcohol consumption (Oktoberfest) did not increase the prevalence of acute pancreatitis.^67^ In healthy young adult volunteers, short-term high alcohol consumption (blood ethanol level at 6 hours, 1.6 ± 0.3 g/L) did not result in any sign of pancreatic injury.^68^^,^^69^ It should be noted that the subjects received a full dinner during alcohol intake in the study.

On the contrary, median 30-month high consumption among continuous drinkers resulted in signs of subclinical pancreatic injury, as detected with increases in serum pancreatic enzyme and pancreatitis-associated protein levels. These levels were highest at 2 days after the discontinuation of alcohol consumption. No clinical signs of pancreatitis, and no increases in leukocytes or C-reactive protein levels, were detected.^69^^,^^70^ Also, the amount of alcohol consumed prior to acute pancreatitis affected the severity of the first, but not the recurrent, episodes of alcoholic pancreatitis.^71^ Thus, long-term high-dose alcohol consumption seems to affect the pancreas, and if acute pancreatitis follows, the severity may be dose dependent. However, such acute pancreatitis is rare. Chronic changes may be protective, as described in this paragraph. In fact, in one study, patients without a history of pancreatobiliary disease who underwent endo-ultrasonography for reasons unrelated to pancreatobiliary disorders, had changes consistent with chronic pancreatitis that increased with age in almost half of patients aged > 60 years.^72^

Two thirds of patients develop clinical symptoms of acute alcoholic pancreatitis during the first or second day after heavy consumption of alcohol, not during the consumption period.^73^ In fact, contrary to patients with alcoholic hepatic emergencies, patients with acute pancreatitis do not usually come to hospital under the influence of alcohol, which is why measuring alcohol levels does not detect the alcohol etiology of pancreatitis. This is also why tests to detect previous long-term high alcohol consumption have been developed, although negative alcohol tests on admission have been paid very little attention from this pathophysiological point of view.^74^^,^^75^

Previously an attempt was made to compare the diets of patients with acute alcoholic pancreatitis and those with alcoholic liver disease. The two groups consumed nutrients similarly, between 2000 and 3000 kcal/day. However, the data were obtained 2 years after acute pancreatitis referring to the period 6 months before the disease, bringing into question the reliability of the findings. Also, recurrent and first episodes were not differentiated.^76^

Finland has one of the highest prevalences of acute alcoholic pancreatitis in the world, but it is decreasing along with decreased alcohol consumption in the country.^77^ The usual history before the first acute alcoholic pancreatitis includes the patient consuming a lot of alcohol long term and without regular normal eating and drinking of water. They are often very dehydrated when admitted to the hospital, partly due to fluid sequestration due to the disease and partly due to the lack of water consumption. During the withdrawal period, the lack of food and water intake may increase the concentrations of substances in bile and pancreatic fluids, promoting sludge or even small stone formation in either or both.^78^^,^^79^ In fact, in some patients, biliary sludge is detected during convalescence from the first acute alcoholic pancreatitis. Only then either gallstone-induced pancreatitis or gallstones plus alcohol-induced pancreatitis is considered. But should one consider those cases as alcohol-induced pancreatitis mediated by gallstones/sludge possibly induced by alcohol consumption? Also in idiopathic pancreatitis, initially undetected sludge has been found in 60% of removed gallbladders.^80^

Conclusion: Fasting and dehydration during long-term periods of heavy alcohol consumption may be important factors predisposing to acute alcoholic pancreatitis. Sludge in pancreatic fluid or bile may develop in some cases, being the predisposing factors in such cases. The presence of sludge, however, does not on its own explain the initiation of acute pancreatitis if without simultaneous pancreas stimulation.

## Effects of Long-Term Alcohol Exposure on Pancreatic Stimulation–Induced Pancreatic Injury

In rats, long-term alcohol consumption alone does not cause pancreatitis but does impair exocrine pancreatic function by decreasing blood and pancreatic cholecystokinin.^81^ This inhibition of secretion by lower cholecystokinin levels results in compensatory up-regulation at the acinar cell level, explaining the relative hyperstimulation after the cessation of alcohol intake and a return to normal diet.^82^

Hyperstimulation by cholecystokinin or a greater physiological level of cholecystokinin stimulus plus alcohol has been used in in vitro models of acute alcoholic pancreatitis with basolateral exocytosis and the formation of autophagosomes.^83^ Chronic ethanol intake does not induce any change in the expression of acinar cell cholecystokinin receptors, whereas cholinergic muscarinic receptors decrease or stay unchanged, but pancreatic acetylcholinesterase activity decreases, thus sensitizing the pancreas to cholinergic stimulus.^32^^,^^84^^,^^85^

Intracellularly, the mechanism by which alcohol alters the stimulated excretion includes the discontinuation of calcium ion pulsation in the endoplasmic reticulum and changes in mitochondrial oxidation.^86^, ^87^, ^88^ Furthermore, cholecystokinin stimulation induces proinflammatory cytokine cascade in alcohol-sensitized pancreas.^89^

Interestingly, a half-century ago, it was already shown that after fasting, feeding results in higher pancreatic protein synthesis activity than without preceding fasting.^90^ Fasting exacerbates experimental acute pancreatitis.^91^ In clinical acute alcoholic pancreatitis, it is not the absolute fasting during long drinking periods, but relative fasting, together with high amounts of alcohol consumed, may be sufficient to drive down pancreatic output. Dehydration may exacerbate nutritional depletion.^79^

Conclusion: Long-term high-dose alcohol consumption, associated with relative fasting and dehydration, may act synergistically and sensitize the pancreas to carbachol and cholecystokinin hyperstimulation during return to normal diet during the withdrawal period. Thus, temporary obstruction may not be necessary in all cases.

## Breakdown of Protection Mechanisms by Alcohol

Observations that long-term ethanol feeding in animals led to the concept that the pancreatic acinar cells develop protective mechanisms to prevent pancreatitis. Investigations of protective mechanisms have been focused on the pathways of the unfolded protein response, also referred to as the endoplasmic reticulum stress pathways.^92^

Alcohol feeding increases the expression and activation of a key transcription factor, X-box binding protein (XBP)-1, which plays a central role in the unfolded protein response. XBP-1 activates numerous cellular pathways that allow cells to tolerate endoplasmic reticulum stress. In the case of alcohol, oxidative metabolites of ethanol induce stress. In fact, the inhibition of the increase of XBP-1 with alcohol feeding results in pancreatitis responses. This finding raises the possibility that genetic and lifestyle factors can promote a pancreatitis response by preventing the adaptive and protective response coming from the XBP-1 transcription factor. As an example, smoking inhibits the XBP-1 protective response, which may account for the additive effect of smoking in alcoholic pancreatitis.^93^ Other factors such as diet, or deficits therein, could act similarly.

## Hypothesis

Figures 1 and 2,^11^ summarize the effects of alcohol and its withdrawal on the pancreas. These lead to the conclusion that acute alcoholic pancreatitis is initiated via the stimulation of secretion against temporary obstruction caused by precipitations in pancreatic fluid or sometimes in bile. The latter may then be referred to as bile sludge–mediated alcoholic pancreatitis, or as relative hyperstimulation without any obstruction. Physiological pancreas stimulation after normal or especially after fatty diet consumption during the withdrawal period forces the secretion against the obstruction. All cases do not need precipitation-induced obstruction, given that fasting and long-term heavy alcohol consumption may down-regulate cholinergic- and cholecystokinin-mediated pancreatic secretion so low that the acini experience relative hyperstimulation after the return to nonalcoholic diet during the withdrawal period. This is mediated by a decrease in pancreatic acetylcholinesterase activity and possibly by down-regulation of muscarinic receptors, whereas cholecystokinin receptors stay unchanged. For the final development of the injury, the deterioration of cell self-defense mechanisms by alcohol is important.

## Impacts on Current Therapy

Contrary to post-ERCP pancreatitis and postoperative pancreatitis, where preventive measures or early treatments may be eligible, there are no therapeutic interventions for the early events of acute alcoholic pancreatitis. The mechanisms hypothesized occur before the patient seeks treatment. Atropine, somatostatin, aprotinin, and related attempts have understandably failed, as have many substances targeted against inflammation.^94^ The best preventive measure is not to consume alcohol, which also protects against recurrences.^3^

## Impacts on Future Studies

• When the avoidance of alcohol fails, other measures to prevent pancreatitis may be attempted, as is the case of alcohol metabolite acetaldehyde–induced gastrointestinal cancers.^95^^,^^96^
• There is a loss in cholecystokinin and cholinergic stimulation during long-term heavy alcohol consumption. This mechanism should be further studied to understand how to maintain their levels in case of alcohol use.
• The mediators of acetylcholinesterase decrease should be understood in attempts to prevent cholinergic stimulus after withdrawal.
• Studying the polymorphism in muscarinic and cholecystokinin A type receptors would give further insight into the differences in individual susceptibilities to pancreatitis.
• Differences in diets during the intake and withdrawal periods between those who developed acute alcoholic pancreatitis and those who did not should be better understood.
• The molecular mechanisms of protective adaptation to alcohol, especially how genetic and/or environmental factors impair protective mechanisms, should be better understood.
• In addition to what has been already studied,^63^ predictive models for the recurrence of pancreatitis due to alcoholism are necessary to identify patients who are vulnerable to progression to chronic pancreatitis when they cannot abstain from alcohol, in order to provide the opportunity to develop robust prevention management around lifestyle and chemoprevention.

## Disclosure Statement

None declared.

## References

[1] Working Group IAP/APA Acute Pancreatitis Guidelines (2013). IAP/APA evidence-based guidelines for the management of acute pancreatitis. Pancreatology.

[2] Johnstone M., Marriott P., Royle T.J., Richardson C.E., Torrance A., Hepburn E., Bhangu A., Patel A., Bartlett D.C., Pinkney T.D., Gallstone Pancreatitis Study Group; West Midlands Research Collaborative (2014). The impact of timing of cholecystectomy following gallstone pancreatitis. Surgeon.

[3] Nordback I., Pelli H., Lappalainen-Lehto R., Järvinen S., Räty S., Sand J. (2009). The recurrence of acute alcohol associated pancreatitis can be reduced: a randomized controlled trial. Gastroenterology.

[4] Apte M.V., Pirola R.C., Wilson J.S. (2016). Pancreapedia: Exocrine Pancreas Knowledge Base.

[5] Pallagi P., Toth E., Görög M., Venglovecz V., Madacsy T., Varga A., Molnar T., Papp N., Szabo V., Kuthy-Sutus E., Molnar R., Ordog A., Borka K., Schnur A., Keri A., Kajner G., Cseko K., Ritter E., Csupor D., Helyes Z., Galbacs G., Szentesi A., Czako L., Rakonczay Z., Takacs T., Maleth J., Hegyi P. (2024). Heavy metals in cigarette smoke strongly inhibit pancreatic ductal function and promote development of chronic pancreatitis. Clin Transl Med.

[6] Zorniak M., Sirtl S., Mayerle J., Beyer G. (2020). What do we currently know about the pathophysiology of alcoholic pancreatitis: a brief review. Visc Med.

[7] Khan J., Pelli H., Lappalainen-Lehto R., Jarvinen S., Sand J., Nordback I. (2009). Helicobacter pylori in alcohol induced acute pancreatitis. Scand J Surg.

[8] Khan J., Nordback I., Seppänen H., Lappalainen-Lehto R., Järvinen S., Oikarinen S., Tauriainen S., Raty S., Hyoty H. (2013). Is alcoholic pancreatitis associated with enteroviral infection?. World J Gastroenterol.

[9] Khan J., Solakivi T., Seppänen H., Lappalainen-Lehto R., Järvinen S., Ronkainen J., Sand J., Nordback I. (2012). Serum lipid and fatty acid profiles are highly changed in patients with alcohol induced acute pancreatitis. Pancreatology.

[10] Voronina S., Chvanov M., De Faveri F., Mayer U., Wileman T., Criddle D., Tepikin A. (2022). Autophagy, acute pancreatitis and the metamorphoses of a trypsinogen-activating organelle. Cells.

[11] Zhou Q., Melton D.A. (2018). Pancreas regeneration. Nature.

[12] Nordback I.H., Clemens J.A., Chacko V.P., Olson J.L., Cameron J.L. (1991). Changes in high-energy phosphate metabolism and cell morphology in four models of acute experimental pancreatitis. Ann Surg.

[13] Chen X., Zhong R., Hu B. (2025). Mitochondrial dysfunction in the pathogenesis of acute pancreatitis. Hepatobiliary Pancreat Dis Int.

[14] Lamsa T., Jin H.T., Nordback P.H., Sand J., Luukkaala T., Nordback I. (2009). Pancreatic injury response is different depending on the method of resecting the parenchyma. J Surg Res.

[15] Laaninen M., Bläuer M., Sand J., Nordback I., Laukkarinen J. (2014). Difference in early activation of NF-[kappa]B and MCP-1 in acinar-cell-rich versus fibrotic human pancreas exposed to surgical trauma and hypoxia. Gastroenterol Res Pract.

[16] Haciahmetoglu T., Ertekin C., Dolay K., Yanar F., Yanar H., Kapran Y. (2008). The effects of contrast agent and intraductal pressure changes on the development of pancreatitis in an ERCP model in rats. Langenbecks Arch Surg.

[17] Zhang D., Man X., Li L., Tang J., Liu F. (2022). Radiocontrast agent and intraductal pressure promote the progression of post-ERCP pancreatitis by regulating inflammatory response, cellular apoptosis, and tight junction integrity. Pancreatology.

[18] Cannon A., Thompson C.M., Bhatia R., Armstrong K.A., Solheim J.C., Kumar S., Surinder Kumar B. (2021). Molecular mechanisms of pancreatic myofibroblast activation in chronic pancreatitis and pancreatic ductal adenocarcinoma. J Gastroenterol.

[19] Srinivasan M.P., Bhopale K.K., Caracheo A.A., Kaphalia L., Loganathan G., Balamurugan A.N., Rastellini C., Kaphalia B.S. (2021). Differential cytotoxicity, ER/oxidative stress, dysregulated AMPKa[alpha] signaling, and mitochondrial stress by ethanol and its metabolites in human pancreatic acinar cells. Alcohol Clin Exp Res.

[20] Ren Z., Yang F., Wang X., Wang Y., Xu M., Frank J.A., Ke Z.-J., Zhang Z., Shi X., Luo J. (2016). Chronic plus binge ethanol exposure causes more severe pancreatic injury and inflammation. Toxicol Appl Pharmacol.

[21] Ren Z., Wang X., Xu M., Yang F., Frank J.A., Ke Z.J., Luo J. (2016). Binge ethanol exposure causes endoplasmic reticulum stress, oxidative stress and tissue injury in the pancreas. Oncotarget.

[22] Zheng M., Li H., Sun L., Brigstock D.R., Gao R. (2021). Interleukin-6 participates in human pancreatic stellate cell activation and collagen I production via TGF-[beta]1/Smad pathway. Cytokine.

[23] Nordback I.H., MacGowan S., Potter J.J., Cameron J.L. (1991). The role of acetaldehyde in the pathogenesis of acute alcoholic pancreatitis. Ann Surg.

[24] Singh D., Negi T.S., Upadhyay G., Choudhuri G. (2015). Polymorphism of alcohol metabolizing gene ADH3 predisposes to development of alcoholic pancreatitis in North Indian population. Front Mol Biosci.

[25] Werner J., Saghir M., Warshaw A.L., Lewandrowski K.B., Laposata M., Iozzo R.V., Carter E.A., Schatz R.J., Fernandez-del Castillo C. (2002). Alcoholic pancreatitis in rats: injury from nonoxidative metabolites of ethanol. Am J Physiol Gastrointest Liver Physiol.

[26] Jakkampudi A., Jangala R., Reddy R., Reddy B., Venkat Rao G., Pradeep R., Reddy D.N., Talukdar R. (2020). Fatty acid ethyl ester (FAEE) associated acute pancreatitis: an ex-vivo study using human pancreatic acini. Pancreatology.

[27] Korsten M.A., Haber P.S., Wilson J.S., Lieber C.S. (1995). The effect of chronic alcohol administration on cerulein-induced pancreatitis. Int J Pancreatol.

[28] Nyborg N.C.B., Kirk R.K., de Boer A.S., Andersen D.W., Bugge A., Wulff B.S., Thorup I., Clausen T.R. (2020). Cholecystokinin-1 receptor agonist induced pathological findings in the exocrine pancreas of non-human primates. Toxicol Appl Pharmacol.

[29] Lam P.P., Cosen Binker L.I., Lugea A., Pandol S.J., Gaisano H.Y. (2007). Alcohol redirects CCK-mediated apical exocytosis to the acinar basolateral membrane in alcoholic pancreatitis. Traffic.

[30] Gronroos J.M., Laine J., Kaila T., Nevalainen T.J. (1994). Chronic alcohol intake and carbachol-induced acute pancreatitis in the rat. Exp Toxicol Pathol.

[31] Liu S., Oguchi Y., Borner J.W., Runge W., Dressel T.D., Goodale R.L. (1990). Increased canine pancreatic acinar cell damage after organophosphate and acetylcholine or cholecystokinin. Pancreas.

[32] Grönroos J.M., Kaila T., Aho H.J., Nevalainen T.J. (1989). Decrease in the number of muscarinic receptors in rat pancreas after chronic alcohol intake. Pharmacol Toxicol.

[33] Grönroos J.M., Kaila T., Hietaranta A.J. (1994). Alcohol, pancreatic muscarinic receptors and acute pancreatitis. Exp Toxicol Pathol.

[34] Sousa L., Boadas J., Kiriakos D., Borges A., Boadas J., Marcano J., Turkali I., De Los Rios M. (2007). Scorpionism due to Tityus neoespartanus (Scorpiones, Buthidae) in Margarita Island, northeastern Venezuela. Rev Soc Bras Med Trop.

[35] Liang T., Dolai S., Xie L., Winter E., Orabi A.I., Karimian N., Cosen-Binger L.I., Huang Y.-C., Thorn P., Cattral M.S., Gaisano H.Y. (2017). Ex vivo human pancreatic slice preparations offer a valuable model for studying pancreatic exocrine biology. J Biol Chem.

[36] Yang X., Yao L., Fu X., Mukherjee R., Xia Q., Jakubowska M.A., Ferdek P.E., Huang W. (2020). Experimental acute pancreatitis models: history, current status, and role in translational research. Front Physiol.

[37] Raty S., Jaakkola M., Karjalainen J., Kuivanen H., Sand J., Nordback I. (1997). The presence of the gallbladder is associated with the severity of acute biliary pancreatitis. Int J Pancreatol.

[38] Perides G., Laukkarinen J.M., Vassileva G., Steer M.L. (2010). Biliary acute pancreatitis in mice is mediated by the Gprotein-coupled cell surface bile acid receptor Gpbar1. Gastroenterology.

[39] Zi Z., Rao Y. (2024). Discoveries of GPR39 as an evolutionarily conserved receptor for bile acids and of its involvement in biliary acute pancreatitis. Sci Adv.

[40] An P., Fan Y., Wang Q., Huang N., Chen H., Sun J., Du Z., Zhang C., Li J. (2024). Cholic acid activation of GPBAR1 does not induce or exacerbate acute pancreatitis but promotes exocrine pancreatic secretion. Biochem Biophys Res Commun.

[41] Lerch M.M., Saluja A.K., Runzi M., Dawra R., Saluja M., Steer M.L. (1993). Pancreatic duct obstruction triggers acute necrotizing pancreatitis in the opossum. Gastroenterology.

[42] Murayama K.M., Samuel I., Toriumi Y., Solomon T.E., Turkelson C.M., Joehl R.J. (1993). Increased circulating cholecystokinin in obstruction-induced acute pancreatitis. I. Bile duct obstruction with and without pancreatic duct obstruction. J Surg Res.

[43] Mizutani S., Miyata M., Izukura M., Tanaka Y., Matsuda H. (1995). Role of bile and trypsin in the release of cholecystokinin in humans. Pancreas.

[44] Chen Y.F., Chey W.Y., Chang T.M., Lee K.Y. (1985). Duodenal acidification releases cholecystokinin. Am J Physiol.

[45] Herzig K.H., Louie D.S., Owyang C. (1994). Somatostatin inhibits CCK release by inhibiting secretion and action of CCK-releasing peptide. Am J Physiol.

[46] Niederau C., Borchard F., Luthen R., Niederau M. (1996). Early development of experimental biliary pancreatitis and its amelioration by CCK-receptor blockade. Hepatogastroenterology.

[47] Chen C.C., Wang S.S., Tsay S.H., Lee F.Y., Wu S.L., Lu R.-H., Chang F.-Y., Lee S.-D. (1998). Effects of high dose octreotide on retrograde bile salt-induced pancreatitis in rats. Peptides.

[48] Rojas-Victoria E.J., Hernández-Ruiz S.I., García-Perdomo H.A. (2024). Effectiveness of the pharmacological therapy to prevent post ERCP acute pancreatitis: a network meta-analysis. Expert Rev Gastroenterol Hepatol.

[49] Räty S., Sand J., Laine S., Harmoinen A., Nordback I. (1999). Cholecystokinin in the early course of acute post-ERCP pancreatitis. J Am Coll Surg.

[50] Räty S., Sand J., Kemppainen E., Laine S., Nordback I. (2000). Cholecystokinin in acute alcoholic and biliary pancreatitis. Int J Pancreatol.

[51] Li J., Wang R., Tang C. (2011). Somatostatin and octreotide on the treatment of acute pancreatitis - basic and clinical studies for three decades. Curr Pharm Des.

[52] Uhl W., Büchler M.W., Malfertheiner P., Beger H.G., Adler G., Gaus W. (1999). A randomised, double blind, multicentre trial of octreotide in moderate to severe acute pancreatitis. Gut.

[53] Nordback I.H., Cameron J.L. (1993). The mechanism of conversion of xanthine dehydrogenase to xanthine oxidase in acute pancreatitis in the canine isolated pancreas preparation. Surgery.

[54] Nordback I.H., Chacko V.P., Cameron J.L. (1994). Induction of anaerobic glucose metabolism during the development of acute pancreatitis. Ann Surg.

[55] Nordback I.H., Clemens J.A., Cameron J.L. (1991). The role of cholecystokinin in the pathogenesis of acute pancreatitis in the isolated pancreas preparation. Surgery.

[56] Nordback I.H., Olson J.L., Chacko V.P., Cameron J.L. (1995). Detailed characterization of experimental acute alcoholic pancreatitis. Surgery.

[57] Aronen A., Aittoniemi J., Huttunen R., Siiki A., Antila A., Sand J., Laukkarinen J. (2024). P-suPAR may reflect the inflammatory response after pancreatic surgery. Pancreatology.

[58] Antila A., Siiki A., Sand J., Laukkarinen J. (2019). Perioperative hydrocortisone treatment reduces postoperative pancreatic fistula rate after open distal pancreatectomy. a randomized placebo-controlled trial. Pancreatology.

[59] Sarles H. (1974). Chronic calcifying pancreatitis--chronic alcoholic pancreatitis. Gastroenterology.

[60] Hegyi P., Rakonczay Z. (2015). The role of pancreatic ducts in the pathogenesis of acute pancreatitis. Pancreatology.

[61] Venglovecz V., Grassalkovich A., Toth E., Ebert A., Gal E., Korsos M.M., Maleth J., Rakonczay Z., Galla Z., Monostori P., Hegyi P. (2024). Restoring CFTR function with Orkambi decreases the severity of alcohol-induced acute pancreatitis. J Physiol.

[62] Kong F., Pan Y., Wu D. (2024). Activation and regulation of pancreatic stellate cells in chronic pancreatic fibrosis: a potential therapeutic approach for chronic pancreatitis. Biomedicines.

[63] Pelli H., Sand J., Laippala P., Nordback I. (2000). Long-term follow-up after the first episode of acute alcoholic pancreatitis: time course and risk factors for recurrence. Scand J Gastroenterol.

[64] Nikkola J., Laukkarinen J., Lahtela J., Seppänen H., Järvinen S., Nordback I., Sand J. (2017). The long-term prospective follow-up of pancreatic function after the first episode of acute alcoholic pancreatitis: recurrence predisposes one to pancreatic dysfunction and pancreatogenic diabetes. J Clin Gastroenterol.

[65] Nikkola J., Rinta-Kiikka I., Räty S., Laukkarinen J., Lappalainen-Lehto R., Järvinen S., Seppanen H., Nordback I., Sand J. (2014). Pancreatic morphological changes in long-term follow-up after initial episode of acute alcoholic pancreatitis. J Gastrointest Surg.

[66] Laaninen M., Bläuer M., Vasama K., Jin H., Raty S., Sand J., Nordback I., Laukkarinen J. (2012). The risk for immediate postoperative complications after pancreaticoduodenectomy is increased by high frequency of acinar cells and decreased by prevalent fibrosis of the cut edge of pancreas. Pancreas.

[67] Phillip V., Huber W., Hagemes F., Lorenz S., Matheis U., Preinfalk S., Schuster T., Lippl F., Saugel B., Schmid R.M. (2011). Incidence of acute pancreatitis does not increase during Oktoberfest but is higher than previously described in Germany. Clin Gastroenterol Hepatol.

[68] Jaakkola M., Sillanaukee P., Suomalainen H., Koivula T., Nordback I. (1992). Effect of a high dose of ethanol on serum pancreatic enzymes in young healthy adults. Am J Gastroenterol.

[69] Nordback I., Jaakkola M., Iovanna J.L., Dagorn J.C. (1995). Increased serum pancreatitis associated protein (PAP) concentration after longterm alcohol consumption: further evidence for regular subclinical pancreatic damage after heavy drinking?. Gut.

[70] Jaakkola M., Frey T., Sillanaukee P., Koivula T., Nordback I. (1994). Acute pancreatic injury in asymptomatic individuals after heavy drinking over the long-term. Hepatogastroenterology.

[71] Jaakkola M., Sillanaukee P., Löf K., Koivula T., Nordback I. (1994). Amount of alcohol is an important determinant of the severity of acute alcoholic pancreatitis. Surgery.

[72] Rajan E., Clain J.E., Levy M.J., Norton I.D., Wang K.K., Wiersema M.J., Sequeiros E.V., Nelson B.J., Jondal M., Kendall R.K., Harmsen W.S., Zinsmeister A.R. (2005). Age-related changes in the pancreas identified by EUS: a prospective evaluation. Gastrointest Endosc.

[73] Nordback I., Pelli H., Lappalainen-Lehto R., Sand J. (2005). Is it long-term continuous drinking or the post-drinking withdrawal period that triggers the first acute alcoholic pancreatitis?. Scand J Gastroenterol.

[74] Jaakkola M., Sillanaukee P., Lof K., Koivula T., Nordback I. (1994). Blood tests for detection of alcoholic cause of acute pancreatitis. Lancet.

[75] Wong N., Gu C., Yadav D., Cote G.A., Oregon Pitt Pancreatology Working Group (2024). Phosphatidylethanol detects occult heavy alcohol use in patients with acute and chronic pancreatitis. Clin Gastroenterol Hepatol.

[76] Wilson J.S., Bernstein L., McDonald C., Tait A., McNeil D., Pirola R.C. (1985). Diet and drinking habits in relation to the development of alcoholic pancreatitis. Gut.

[77] Sand J., Valikoski A., Nordback I. (2009). Alcohol consumption in the country and hospitalizations for acute alcohol pancreatitis and liver cirrhosis during a 20-year period. Alcohol Alcohol.

[78] Johansson K., Sundström J., Marcus C., Hemmingsson E., Neovius M. (2014). Risk of symptomatic gallstones and cholecystectomy after a very-low-calorie diet or low-calorie diet in a commercial weight loss program: 1-year matched cohort study. Int J Obes (Lond).

[79] Coskun E., Yang A.L. (2024). Marathon pancreatitis: a case of acute pancreatitis caused by distance running. BMJ Case Rep.

[80] Räty S., Pulkkinen J., Nordback I., Sand J., Victorzon M., Gronroos J., Helminen H., Kuusanmaki P., Nordstrom P., Paajanen H. (2015). Can laparoscopic cholecystectomy prevent recurrent idiopathic acute pancreatitis?: a prospective randomized multicenter trial. Ann Surg.

[81] Li J., Guo M., Hu B., Liu R., Wang R., Tang C. (2008). Does chronic ethanol intake cause chronic pancreatitis?: evidence and mechanism. Pancreas.

[82] Deng X., Wood P.G., Eagon P.K., Whitcomb D.C. (2005). Rapid adaptation of pancreatic exocrine function to short-term alcohol feeding in rats. Pancreatology.

[83] Dolai S., Takahashi T., Qin T., Liang T., Xie L., Kang F., Miao Y.-F., Xie H., Kang Y., Manuel J., Winter E., Roche P.A., Cattral M.S., Gaisano H.Y. (2021). Pancreas-specific SNAP23 depletion prevents pancreatitis by attenuating pathological basolateral exocytosis and formation of trypsin-activating autolysosomes. Autophagy.

[84] Li J., Zhou C., Wang R., Liu R., Huang Z., Tang C. (2010). Irreversible exocrine pancreatic insufficiency in alcoholic rats without chronic pancreatitis after alcohol withdrawal. Alcohol Clin Exp Res.

[85] Lugea A., Gong J., Nguyen J., Nieto J., French S.W., Pandol S.J. (2010). Cholinergic mediation of alcohol-induced experimental pancreatitis. Alcohol Clin Exp Res.

[86] Petersen O.H., Sutton R. (2006). Ca2+ signalling and pancreatitis: effects of alcohol, bile and coffee. Trends Pharmacol Sci.

[87] Gonzalez A., Pariente J.A., Salido G.M. (2008). Ethanol impairs calcium homeostasis following CCK-8 stimulation in mouse pancreatic acinar cells. Alcohol.

[88] Manko B.O., Bilonoha O.O., Voloshyn D.M., Zub A.M., Ivasechko I.I., Manko V.V. (2021). Pyruvate and glutamine define the effects of cholecystokinin and ethanol on mitochondrial oxidation, necrosis, and morphology of rat pancreatic acini. Pancreas.

[89] Pandol S.J., Periskic S., Gukovsky I., Zaninovic V., Jung Y., Zong Y., Solomon T.E., Gukovskaya A.S., Tsukamoto H. (1999). Ethanol diet increases the sensitivity of rats to pancreatitis induced by cholecystokinin octapeptide. Gastroenterology.

[90] Webster P.D., Singh M., Tucker P.C., Black O. (1972). Effects of fasting and feeding on the pancreas. Gastroenterology.

[91] Souza M.L., Ariga S., Barbeiro D.F., Machado M.A., Machado M.C., Souza H.P. (2024). Fasting increases the severity of acute pancreatitis in a mouse model:implications for preoperative interventions to reduce complications of pancreatic surgery. Arq Gastroenterol.

[92] Lugea A., Tischler D., Nguyen J., Gong J., Gukovsky I., French S.W., Gorelick F.S., Pandol S.J. (2011). Adaptive unfolded protein response attenuates alcohol-induced pancreatic damage. Gastroenterology.

[93] Lugea A., Gerloff A., Su H.Y., Xu Z., Go A., Hu C., French S.W., Wilson J.S., Apte M.V., Waldron R.T., Pandol S.J. (2017). The combination of alcohol and cigarette smoke induces endoplasmic reticulum stress and cell death in pancreatic acinar cells. Gastroenterology.

[94] Hey-Hadavi J., Velisetty P., Mhatre S. (2023). Trends and recent developments in pharmacotherapy of acute pancreatitis. Postgrad Med.

[95] Nieminen M.T., Salaspuro M. (2018). Local acetaldehyde-an essential role in alcohol-related upper gastrointestinal tract carcinogenesis. Cancers (Basel).

[96] Hellstrom P.M., Hendolin P., Kaihovaara P., Kronberg L., Meierjohann A., Millerhovf A., Paloheimo L., Sundelin H., Syrjänen K., Webb D.-L., Salaspuro M. (2017). Slow-release L-cysteine capsule prevents gastric mucosa exposure to carcinogenic acetaldehyde: results of a randomised single-blinded, cross-over study of Helicobacter-associated atrophic gastritis. Scand J Gastroenterol.

